# Retrospective, single-center assessment of a pharmacist-led anti-methicillin-resistant *Staphylococcus aureus* (MRSA) therapy bundle to enhance antimicrobial stewardship

**DOI:** 10.1017/ash.2025.10164

**Published:** 2025-10-06

**Authors:** Jeyma Fernandez, Alice Margulis Landayan, Jorge Murillo, Grace Hoffman, Stephen Breazeale, Timothy P. Gauthier

**Affiliations:** 1 Department of Pharmacy, South Miami Hospital, South Miami, FL, USA; 2 Department of Medicine, South Miami Hospital, South Miami, FL, USA; 3 Baptist Health Academics, Baptist Health South Florida, Miami, FL, USA; 4 Clinical Pharmacy Enterprise, Baptist Health South Florida, Miami, FL, USA

## Introduction

Methicillin-resistant *Staphylococcus aureus* (MRSA) is classified by the Centers for Disease Control and Prevention (CDC) as a serious antimicrobial resistance threat.^1^ Unnecessary use of anti-MRSA therapy can lead to an increase in resistance development and adverse drug effects.^2^ Pharmacist-led interventions can facilitate de-escalation and discontinuation of broad-spectrum antibiotics and improve outcomes.^3,4^ The use of MRSA nasal polymerase chain reaction (PCR) testing can support de-escalation and discontinuation of unnecessary anti-MRSA therapy.^5–8^ The aim of this quality improvement project was to evaluate the impact of a new pharmacist-led anti-MRSA therapy bundle on pharmacist interventions and anti-MRSA therapy utilization.

## Methods

This retrospective quality improvement project evaluated the impact of a pharmacist-led anti-MRSA therapy bundle at a 453-bed community hospital comparing January through March 2024 (pre-implementation) vs January through March 2025 (post-implementation). The anti-MRSA therapy bundle consisted of the reassessment and creation of additional VigiLanz® (an electronic clinical surveillance program) alerts, expanding MRSA nasal PCR screening from solely pulmonary to nonpulmonary indications as well, creation of a “MRSA Antibiotic Time-Out” document with pharmacist education, and use of a report identifying active orders for daptomycin, linezolid, and vancomycin. Designated pharmacists (a pharmacist resident, an antimicrobial stewardship pharmacist, and a critical care pharmacist) assessed therapy for appropriateness and opportunities for intervention. Interventions were completed either per-protocol without the need to contact providers (eg, IV to PO) or per provider approval (eg, antimicrobial change) by all clinical pharmacists.

Adult patients who were admitted to the hospital with orders for scheduled daptomycin intravenous (IV), linezolid IV/ by mouth (PO), or vancomycin IV were included. Exclusion criteria consisted of orders for “pre-op,” “on-call,” or “once” frequencies, indication of surgical prophylaxis or group B *Streptococcus* prophylaxis, patients on chronic suppressive or prophylactic antimicrobial therapy, hospice care and comfort measures only, incarcerated, and pregnant patients.

The primary end point was the number of patients who received at least one pharmacist intervention associated with daptomycin IV, linezolid IV/PO, or vancomycin IV related to anti-MRSA therapy de-escalation, discontinuation, or optimization. Secondary end points included the total number of pharmacist interventions related to anti-MRSA therapy de-escalation, discontinuation, or optimization, pharmacist antibiotic intervention, intervention acceptance rate, VigiLanz® adult hospital-wide inpatient days of therapy per 1 000 patient days, hospital length of stay, incidence of medication-related adverse drug events, and 30-day all-cause mortality rate.

Statistical analysis included χ^2^ test or Fisher’s exact for categorical data and Mann–Whitney U test for continuous data. Calculated minimum sample size was 148 patients (74 patients in each group) based on expected primary outcome incidence of 30% pre-implementation vs 50% post-implementation, with a power of 80%. Statistical significance was determined using *P* < .05. This project was deemed exempt from Institutional Review Board review.

Further details regarding the methods and results are provided in the Supplementary Material.

## Results

For outcome analysis, 217 patients were screened for inclusion, with 100 patients included in each arm. The most common reasons for exclusion were “once” frequencies and comfort measures. The number of patients who received at least one pharmacist intervention was 40% pre-implementation compared to 73% post-implementation; +33% [95% CI; 20, 40], *P* < .0001. Baseline and infection characteristics for both groups are listed in Table 1.

**Table 1.** Baseline and infection characteristics

Characteristics	Pre-Implementation (*N* = 100)	Post-Implementation (*N* = 100)	*P*-value
Median age (IQR), years	72 (63-82)	62 (50-76)	<0.001
Male gender, *n* (%)	53 (53)	49 (49)	0.57
Median weight (IQR), kg	80 (67-90)	76 (65-88)	0.66
Antimicrobial allergy, *n* (%)^1^	24 (24)	27 (27)	0.62
Penicillin	18 (18)	14 (14)	0.44
Cephalosporin	2 (2)	6 (6)	0.27
Sulfa	5 (5)	8 (8)	0.39
Other	6 (6)	8 (8)	0.57
Admission to intensive care unit, *n* (%)	27 (27)	23 (23)	0.51
Most common MRSA risk factors, *n* (%)^2^			
Recent hospitalization	25 (25)	27 (27)	0.75
Prior MRSA infection	11 (11)	9 (9)	0.64
Recent IV antibiotic use	7 (7)	9 (9)	0.60
Infection types, *n* (%)^3^			
** **Bacteremia	5 (5)	7 (7)	0.55
** **Osteomyelitis	3 (3)	11 (11)	0.05
** **Pulmonary infection	24 (24)	11 (11)	0.02
Community-acquired	5 (5)	2 (2)	0.44
Hospital-acquired	19 (19)	7 (7)	0.01
Ventilator-acquired	0 (0)	2 (2)	0.49
** **SSTI	42 (42)	55 (55)	0.07
Non-purulent	22 (22)	14 (14)	0.14
Purulent	20 (20)	41 (41)	0.001
** **Other infection types	31 (31)	23 (23)	0.20
MRSA positive culture from any site, n (%)	9 (9)	15 (15)	0.19
Empirical vs culture-driven initiated gram-positive coverage, *n* (%)			
** **Empirical	97 (97)	94 (94)	0.50
** **Culture-driven	3 (3)	6 (6)	0.50

IQR: interquartile range; Kg: kilogram; MRSA: methicillin-resistant *Staphylococcus aureus*; SSTI: skin and soft tissue infection

^1^ Patients may have allergy to more than one antibiotic.

^2^ Patients may have more than one MRSA risk factor.

^3^ Patients may have more than one infection type.

Compared to the pre-implementation group, the post-implementation group had significantly higher rates of antimicrobial de-escalation (4% vs 14%; +10% [95% CI; 2.2, 17.8], *P* = .02), anti-MRSA agent change (8% vs 21%; +13% [95% CI; 3.4, 22.6], *P* = .009), IV to PO conversion (0% vs 6%; +6% [95% CI; 1.3, 10.7], *P* = .03), and MRSA PCR ordering (5% vs 15%; +10% [95% CI; 1.8, 18.2], *P* = .02). There were no statistically significant differences between antimicrobial discontinuation (25% vs 30%; +5% [95% CI; -7.4, 17.4], *P* = .43), dose optimization (2% vs 2%; 0% [95% CI; -3.9, 3.9], *P* = 1.00), or duration of therapy (4% vs 12%; +8% [95% CI; .6, 15.4], *P* = .07). Although hospital-wide adult inpatient days of therapy per 1 000 patient days was 196 vs 205 (*P* = .62) for the target antibiotics overall, a decrease was observed in vancomycin days of therapy (122 vs 87, *P* = .04). Linezolid and daptomycin days of therapy were 52 vs 68 (*P* = .09) and 22 vs 50 (*P* = .05), respectively. Hospital length of stay was 7 days for both groups; +.18% [95% CI; -3.05, 3.41], *P* = .35. Incidence of medication-related adverse drug events and 30-day all-cause mortality were 0% in both groups (*P* = 1.00). Overall, there was a total of 48 pharmacist interventions pre-implementation and 100 post-implementation; +0.51% [95% CI; 0.31, 0.71].

An intervention acceptance rate of 93% was observed in the post-implementation group, with 14% of interventions completed per-protocol.

Extending beyond the 100 patients reviewed for outcome analysis, in total during the active intervention period 182 patients were assessed for appropriate anti-MRSA therapy with 134 patients receiving at least one intervention and a total of 175 interventions completed.

## Discussion

Our pharmacist-led anti-MRSA therapy bundle increased pharmacist interventions for de-escalation, discontinuation, and optimization of anti-MRSA therapy, with high provider acceptance.

The bundle approach aligns with the World Health Organization’s recommendation to use multimodal strategies, such as bundles to improve clinical processes and patient outcomes.^9^ As focusing on a single intervention has not proven effective in achieving sustained behavioral changes, the bundle approach was used in this project to further enhance outcomes related to anti-MRSA therapy. For example, we did not find MRSA nares screening to be productive for non-pulmonary infections so in turn emphasized this less in the latter portion of the post-period.

The increase in de-escalation of anti-MRSA therapy observed is supported by previous work in which pharmacist involvement and education effectively reduce unnecessary antimicrobial use and ensure more targeted antimicrobial therapy.^10^

This study is limited by its retrospective single-center design, short study time frame, and limited use of MRSA PCR testing for nonpulmonary infections. Additionally, capacity constraints may affect the external validity of the findings, as successful implementation relies on a multidisciplinary team. Although bundled interventions are recommended and have been shown to improve patient outcomes, they make it difficult to determine which individual component had the greatest impact.

Pharmacist-driven anti-MRSA therapy bundles have the potential to positively influence patient care and should be considered a valuable tool within antimicrobial stewardship programs.

## Supporting information

10.1017/ash.2025.10164.sm001 Fernandez et al. supplementary material Fernandez et al. supplementary material

## Supplementary material

The supplementary material for this article can be found at https://doi.org/10.1017/ash.2025.10164.

## References

[1] Antimicrobial Resistance Threats in the United States, 2021-2022. Centers for Disease Control and Prevention.

[2] Liu C , Bayer A , Cosgrove SE , et al. IDSA guidelines for the treatment of Methicillin-resistant *Staphylococcus aureus* (MRSA) in adults and children. Clin Infect Dis 2011;55:e18–e55. 10.1093/cid/ciq146 21208910

[3] Core Elements of Hospital Antibiotic Stewardship Programs. Centers for Disease Control and Prevention. 10.1093/cid/ciu542 PMC6521960 25261548

[4] Dighriri IM , Alnomci BA , Aljahdali MM , et al. The role of clinical pharmacists in Antimicrobial Stewardship Programs (ASP): a systematic review. Cereus 2023;15:1–10. 10.7759/cureus.50151 PMC10771624 38186441

[5] Dangerfield B , Chung A , Webb B , Seville MT. Predictive value of Methicillin-resistant *Staphylococcus aureus* (MRSA) nasal swab PCR assay for MRSA pneumonia. Antimicrob Agents Chemother 2014;58:859–864. 24277023 10.1128/AAC.01805-13 PMC3910879

[6] Harb G , Hopkins T , Yang L , et al. Clinical utility of Methicillin-resistant *Staphylococcus aureus* nasal PCR to streamline antimicrobial use in treatment of diabetic foot infection with or without osteomyelitis. BMC Infect Dis 2023;297:1–6. 10.1186/s12879-023-08248-2 PMC10163799 37147579

[7] Stramel S , Buckley V , Tran MC , Price T. MRSA nasal screening predictive values assessment in patients with osteomyelitis. Antimicrob Stewardsh Healthc Epidemiol 2024;140:1–3. 10.1017/ash.2024.368 PMC11427970 39346656

[8] Noeldner HM , Bliek ZJ , Jones NE , et al. Clinical utility of methicillin-resistant *Staphylococcus aureus* nasal polymerase chain reaction (PCR) assays beyond respiratory infections. Antimicrob Stewardsh Healthc Epidemiol 2022;2:1–3. 10.1017/ash.2022.256 PMC9726598 36483346

[9] Guidelines on Core Components of Infection Prevention and Control Programmes at the National and Acute Health Care Facility Level. World Health Organization website. https://iris.who.int/handle/10665/251730. Published 2016. Accessed May 17, 2025. 27977095

[10] Uda A , Ebisawa K , Sakon H , et al. Sustained improvements in antimicrobial therapy and clinical outcomes following a pharmacists-led Antimicrobial Stewardship Intervention: uncontrolled before-after study. J Clin Med 2022;11:1–11. 10.3390/jcm11030566 PMC8837014 35160018

