# Supplementary material for: Retrospective, single-center assessment of a pharmacist-led anti-methicillin-resistant Staphylococcus aureus (MRSA) therapy bundle to enhance antimicrobial stewardship
Source: Antimicrob Steward Healthc Epidemiol. 2025 Oct 6;5(1):e248. doi: 10.1017/ash.2025.10164 (PMC12509146; doi:10.1017/ash.2025.10164)
Supplement: Fernandez et al. supplementary material [file S2732494X25101642sup001.docx]

Supplementary Material

**Supplementary Table 1: Definitions**

| Term | Definition |
| --- | --- |
| Anti-methicillin-resistant *Staphylococcus aureus* (MRSA) therapy change | Choosing an alternative anti-MRSA agent for those who require continuation of MRSA coverage (i.e. vancomycin changed to linezolid) |
| De-escalation | Narrowing to a less broad antimicrobial agent based on cultures or type of infection (i.e. vancomycin changed to cefazolin when MRSA coverage not indicated) |
| Discontinuation | Stopping unnecessary antimicrobial agent based on patient presentation, type of infection, and cultures (i.e. MRSA nares negative and vancomycin discontinued) |
| Dose optimization | Adjusting antimicrobial agent dose (i.e. adjust daptomycin from 4 mg/kg to 6 mg/kg to account for infection type) |
| Non-pulmonary indications | Any indication outside of the respiratory tract (i.e. diabetic foot infection, skin and soft tissue infection, osteomyelitis) |

**Supplementary Figure 1: Study Implementation and Duration**

**January 2025**

- Post-implementation period
- VigiLanz® alert go-live
- Data collection

**January – March 2024**

- Pre-implementation period

**December 2024**

- Creation of “MRSA Antibiotic Time-Out Tool”
- Creation of VigiLanz® alerts
- Pharmacist in-service

**March 2025**

- End of data collection

**Supplementary Figure 2: Patient Screening and Selection Flowchart for Pre/Post Comparison:**

Total Patients Screened (n=217)

**Supplementary Table 2: Characteristics of Interventions Performed Pre/Post MRSA Bundle Implementation:**

Excluded

Comfort Measures

(n=2)

Excluded

Group B *Streptococcus* Prophylaxis

(n=1)

Comfort Measures

(n=4)

“Once” Frequency

(n=10)

**Post-Implementation** Patients Screened (n=102)

**Pre-Implementation** Patients Screened (n=115)

Included

(n=100)

Included

(n=100)

| Outcome | Pre-Implementation  (N=100) | Post-Implementation  (N=100) | P-value |
| --- | --- | --- | --- |
| Pharmacist antibiotic intervention detail*  Antimicrobial de-escalation, n (%)  Antimicrobial discontinuation, n (%)  Anti-MRSA therapy agent change, n (%)  Dose optimization, n (%)  Duration of therapy, n (%)  Intravenous (IV) to by mouth (PO), n (%)  MRSA PCR ordered, n (%) | 4 (4)  25 (25)  8 (8)  2 (2)  4 (4)  0 (0)  5 (5) | 14 (14)  30 (30)  21 (21)  2 (2)  12 (12)  6 (6)  15 (15) | **0.02**  0.43  **0.009**  1.00  0.07  **0.03**  **0.02** |

^*^Each patient may have more than one intervention
